# Plasma acylcarnitines and risk of lower-extremity functional impairment in older adults: a nested case–control study

**DOI:** 10.1038/s41598-021-82912-y

**Published:** 2021-02-08

**Authors:** Francisco Félix Caballero, Ellen A. Struijk, Alberto Lana, Antonio Buño, Fernando Rodríguez-Artalejo, Esther Lopez-Garcia

**Affiliations:** 1 Department of Preventive Medicine and Public Health, School of Medicine, Universidad Autónoma de Madrid, C/ Arzobispo Morcillo, s/n, 28029 Madrid, Spain; 2 IdiPaz (Instituto de Investigación Sanitaria Hospital Universitario La Paz), Madrid, Spain; 3 CIBERESP (CIBER of Epidemiology and Public Health), Madrid, Spain; 4 Department of Medicine. School of Medicine and Health Sciences, Universidad de Oviedo/ISPA, Oviedo, Spain; 5 Department of Laboratory Medicine, La Paz University Hospital, Madrid, Spain; 6 IMDEA-Food Institute. CEI UAM+CSIC, Madrid, Spain

**Keywords:** Metabolomics, Prognostic markers

## Abstract

Elevated concentrations of acylcarnitines have been associated with higher risk of obesity, type 2 diabetes and cardiovascular disease. The aim of the present study was to assess the association between L-carnitine and acylcarnitine profiles, and 2-year risk of incident lower-extremity functional impairment (LEFI). This case–control study is nested in the Seniors-ENRICA cohort of community-dwelling older adults, which included 43 incident cases of LEFI and 86 age- and sex- matched controls. LEFI was assessed with the Short Physical Performance Battery. Plasma L-carnitine and 28 acylcarnitine species were measured. After adjusting for potential confounders, medium-chain acylcarnitines levels were associated with 2-year incidence of LEFI [odds ratio per 1-SD increase: 1.69; 95% confidence interval: 1.08, 2.64; *p* = 0.02]. Similar results were observed for long-chain acylcarnitines [odds ratio per 1-SD increase: 1.70; 95% confidence interval: 1.03, 2.80; *p* = 0.04]. Stratified analyses showed a stronger association between medium- and long-chain acylcarnitines and incidence of LEFI among those with body mass index and energy intake below the median value. In conclusion, higher plasma concentrations of medium- and long-chain acylcarnitines were associated with higher risk of LEFI. Given the role of these molecules on mitochondrial transport of fatty acids, our results suggest that bioenergetics dysbalance contributes to LEFI.

## Introduction

Metabolomics profiling is a strategy to identify low-weight molecules that are able to characterize the metabolic fingerprint associated with the early stages of disease^1^. This fingerprint allows for assessing multiple metabolic processes occurring in different tissues at a particular time^2^. Although some efforts have been done to disentangle the metabolomics profiling associated with unhealthy aging^3^, low muscle mass^4^, frailty^5,6^, and most recently, decline in gait speed^7,8^, most research was based on cross-sectional designs, showed heterogeneous results, and none but one^7^ study has considered the impact of lifestyles on the identified biological pathways.

Carnitine is a naturally occurring compound found in mammalian species and has an important role in skeletal muscle bioenergetics^9^. The endogenous carnitine pool is comprised of L-carnitine (levocarnitine) and different short-, medium- and long-chain acylcarnitines. Both, L-carnitine and acylcarnitines are involved in mitochondrial transport of fatty acids and are relevant agents for normal mitochondrial function^10^. However, elevated concentrations of these metabolites have been associated with higher risk of obesity^11^, type 2 diabetes^12,13^ and cardiovascular disease^14^. In fact, in a small cross-sectional study, higher plasma concentrations of medium- and long-chain acylcarnitines were correlated with lower levels of physical performance^15^. Another study, with information about self-reported incident mobility disability found an association between four metabolites of carnitine and higher risk of this outcome^16^.

Since muscle deterioration and sarcopenia share some biological pathways with metabolic diseases and cardiovascular disease, including insulin resistance and low-grade inflammation, we hypothesized that higher plasma concentrations of acylcarnitine species could be predictive of impaired physical functioning. A performance-based measure of lower-extremity function, as evaluated with the Short Physical Performance Battery, is an optimal measure of physical function because integrates the work of multiple organ systems, including the heart, lungs, circulatory, nervous, and musculoskeletal systems, as well as enough energy reserves and capacity of movement control. In addition, this test has shown to predict disability^17^, hospitalization and mortality in older adults^18,19^. Therefore, the aim of the present study was to assess the association of L-carnitine and acylcarnitine profiles with incident lower-extremity functional impairment (LEFI), by using a case–control design nested in a cohort of community-dwelling old adults.

## Results

The mean age of the study participants was 72.4 years (SD: 4.8), with a 57.8% of women. The average reduction in the SPPB global score among cases was 3.9 points (SD = 2.1); by contrast, controls kept an optimum level of function, with a slight mean increase of 0.5 points (SD = 1.6) in the score between 2015 and 2017. Compared to controls, cases showed more frequent obesity (37.2% vs. 21.2%, *p* = 0.08) and osteomuscular disease (60.5% vs. 43.5%, *p* = 0.07), and a lower level of physical activity (57.6 ± 33.1 vs. 68.9 ± 36.2 METs h/wk, *p* = 0.09) (Table 1).

**Table 1:** Baseline characteristics of participants in the Seniors-ENRICA nested case–control substudy.

	Case group (*n* = 43)	Control group (*n* = 85)	*p*
**Educational attainment, n (%)**			
Primary education or less	24 (55.8)	35 (41.2)	
Secondary school	8 (18.6)	27 (31.8)	0.21
University	11 (25.6)	23 (27.1)	
**BMI, n (%)**			
< 25 kg/m^2^	13 (30.2)	23 (27.0)	
25–30 kg/m^2^	14 (32.6)	44 (51.8)	0.08
≥ 30 kg/m^2^	16 (37.2)	18 (21.2)	
Alcohol intake, g/d	6.2 ± 11.6	7.6 ± 11.2	0.52
Energy intake, kcal/d	1915 ± 397	2014 ± 453	0.23
Physical activity, METs h/wk	57.6 ± 33.1	68.9 ± 36.2	0.09
**Chronic conditions, n (%)**			
Diabetes	5 (11.6)	8 (9.4)	0.70
Cardiovascular disease	2 (4.7)	4 (4.7)	0.99
Osteomuscular disease	26 (60.5)	37 (43.5)	0.07

Mean ± SD for continuous variables and *n* (%) for categorical variables. Unpaired *t*-test for continuous variables and chi-squared test for categorical variables were used for statistical comparison.

A fully description of the L-carnitine and acylcarnitine species levels is provided in Table 2, where a trend towards higher levels of acylcarnitines in cases can be observed. Higher levels of metabolites were found in the case group for C16 (*p* = 0.04) and C16:1 (*p* = 0.03). The same trend was observed in other acylcarnitine species, especially in the case of C6 (*p* = 0.06) and C14 (*p* = 0.05).

**Table 2:** Geometric means (95% confidence interval) of L-carnitine and acylcarnitines levels (µM) in case and control groups.

	Case group (*n* = 43)	Control group (*n* = 85)	*p*
L-carnitine	46.7 (43.4, 50.3)	47.2 (44.9, 49.5)	0.83
C2	9.1 (8.1, 10.2)	9.0 (8.3, 9.7)	0.86
C3 (10^–1^)	4.5 (3.9, 5.2)	4.7 (4.2, 5.2)	0.65
C4 (10^–1^)	1.1 (0.9, 1.3)	1.1 (1.0, 1.2)	0.93
C5 (10^–2^)	5.7 (5.0, 6.5)	5.2 (4.8, 5.7)	0.26
C5DC (10^–2^)	0.9 (0.8, 1.1)	0.9 (0.8, 1.0)	0.42
C6 (10^–2^)	3.7 (3.0, 4.5)	3.1 (2.8, 3.4)	0.06
C8 (10^–1^)	1.6 (1.3, 2.0)	1.3 (1.2, 1.5)	0.19
C8:1 (10^–2^)	2.7 (2.2, 3.4)	2.3 (2.0, 2.6)	0.16
C10 (10^–1^)	3.8 (3.0, 4.7)	3.1 (2.7, 3.6)	0.16
C10:1 (10^–1^)	1.1 (0.9, 1.4)	1.0 (0.9, 1.1)	0.19
C12 (10^–1^)	1.1 (0.9, 1.4)	0.9 (0.8, 1.0)	0.10
C12:1 (10^–2^)	5.0 (4.0, 6.1)	4.0 (3.5, 4.6)	0.08
C12–OH (10^–2^)	0.6 (0.5, 0.7)	0.5 (0.4, 0.6)	0.29
C14 (10^–2^)	1.8 (1.6, 2.2)	1.6 (1.5, 1.7)	0.05*
C14:1 (10^–2^)	5.6 (4.6, 6.8)	4.8 (4.2, 5.4)	0.15
C14-OH (10^–3^)	2.8 (2.4, 3.2)	2.7 (2.5, 3.0)	0.84
C16 (10^–2^)	8.6 (7.5, 9.9)	7.5 (7.0, 8.0)	0.04*
C16:1 (10^–2^)	2.3 (2.0, 2.8)	1.9 (1.8, 2.1)	0.03*
C16-OH (10^–3^)	1.0 (0.8, 1.2)	0.9 (0.8, 1.0)	0.45
C18 (10^–2^)	3.3 (3.0, 3.8)	3.2 (3.0, 3.4)	0.45
C18:1 (10^–1^)	2.5 (2.1, 2.9)	2.2 (2.0, 2.4)	0.15
C18:2 (10^–2^)	7.0 (5.8, 8.5)	6.2 (5.7, 6.9)	0.21
C18:2–OH (10^–3^)	1.6 (1.3, 2.0)	1.7 (1.5, 1.9)	0.78
C20 (10^–3^)	3.0 (2.7, 3.3)	2.9 (2.7, 3.1)	0.75
C20:1 (10^–2^)	0.9 (0.7, 1.0)	0.8 (0.7, 0.9)	0.34
C20:2 (10^–3^)	3.8 (3.2, 4.6)	3.3 (3.0, 3.6)	0.11
C20:3 (10^–3^)	3.7 (3.1, 4.5)	3.1 (2.7, 3.5)	0.10
C20:4 (10^–3^)	3.8 (3.0, 3.9)	3.4 (3.0, 3.9)	0.33

*p* values were calculated with an unpaired t-test over the log-transformed L-carnitine and acylcarnitines values.

*p < 0.05.

No association was found between levels of L-carnitine and short-chain acylcarnitines, and LEFI (Table 3). However, a significant association was observed between medium-chain acylcarnitines and a higher risk of LEFI, in models adjusted for education, body mass index (BMI), alcohol, energy intake and presence of chronic conditions [OR per 1-SD increase: 1.69; 95% CI: 1.08, 2.64; *p* = 0.02]; results in the same direction were also observed for long-chain acylcarnitines [OR per 1-SD increase: 1.70; 95% CI: 1.03, 2.80; *p* = 0.04]. The additional adjustment for physical activity somewhat weakened the association, although in the case of medium-chain acylcarnitines remained statistically significant [OR per 1-SD increase: 1.62; 95% CI: 1.02, 2.58; *p* = 0.04].

**Table 3:** Conditional logistic regression models for the association between L-carnitine and acylcarnitine scores, and 2-year incidence of lower-extremity functional impairment.

	Odds ratio (95% confidence interval) per 1-SD increase	*p*
**L-carnitine**		
Model 1	0.97 (0.67, 1.41)	0.89
Model 2	0.91 (0.60, 1.41)	0.68
Model 3	0.91 (0.58, 1.42)	0.68
**Short-chain acylcarnitines**		
Model 1	1.37 (0.93, 2.01)	0.12
Model 2	1.45 (0.92, 2.28)	0.11
Model 3	1.51 (0.94, 2.42)	0.09
**Medium-chain acylcarnitines**		
Model 1	1.39 (0.95, 2.04)	0.09
Model 2	1.69 (1.08, 2.64)	0.02*
Model 3	1.62 (1.02, 2.58)	0.04*
**Long-chain acylcarnitines**		
Model 1	1.46 (0.97, 2.18)	0.07
Model 2	1.70 (1.03, 2.80)	0.04*
Model 3	1.61 (0.97, 2.67)	0.07

Inverse normal transformation was applied to raw values of L-carnitine and acylcarnitines. Acylcarnitines were summed to calculate short-, medium-, and long-chain scores.

Model 1 was a crude model, without adjusting for any covariate.

Model 2 was adjusted for level of education (primary education or less, secondary school, university), BMI (< 25, 25–30, ≥ 30 kg/m^2^), alcohol intake (tertiles), energy intake (tertiles), diabetes, cardiovascular disease, and osteomuscular disease.

Model 3 was additionally adjusted for physical activity (tertiles).

*p < 0.05.

Stratified analyses showed a stronger association between medium- and long-chain acylcarnitines and incidence of LEFI among those with BMI and energy intake below the median value (Table 4), although results did not vary across other strata.

**Table 4:** Conditional logistic regression models for the association between L-carnitine and acylcarnitine scores and 2-year incidence of lower-extremity functional impairment, by categories of adherence to the Mediterranean diet, BMI, alcohol and total energy intake (below and above the median).

	L-carnitine	Short-chain acylcarnitines	Medium-chain acylcarnitines	Long-chain acylcarnitines
**Adherence to Mediterranean Diet**				
< 8.0 points in MEDAS	0.88 (0.40, 1.93)	1.68 (0.72, 3.91)	1.56 (0.59, 4.12)	1.77 (0.78, 4.02)
≥ 8.0 points in MEDAS	1.00 (0.55, 1.85)	1.62 (0.75, 3.48)	1.41 (0.72, 2.75)	1.44 (0.60, 3.42)
*p*-value for interaction	0.80	0.95	0.81	0.88
**BMI**				
< 26.9 kg/m^2^	0.72 (0.37, 1.38)	1.38 (0.68, 2.82)	2.15 (1.05, 3.44)*	1.50 (0.74, 3.07)
≥ 26.9 kg/m^2^	0.96 (0.52, 1.80)	1.44 (0.79, 2.64)	1.01 (0.52, 1.95)	1.62 (0.79, 3.34)
*p*-value for interaction	0.35	0.45	0.27	0.93
**Alcohol intake**				
< 1.3 g/day	0.57 (0.28, 1.19)	1.45 (0.74, 2.84)	2.11 (0.96, 4.63)	2.18 (0.95, 5.00)
≥ 1.3 g/day	1.48 (0.74, 2.97)	1.95 (0.88, 4.31)	1.26 (0.62, 2.54)	1.29 (0.61, 2.73)
*p*-value for interaction	0.07	0.26	0.73	0.90
**Energy intake**				
< 1923 kcal/day	1.13 (0.58, 2.20)	1.71 (0.87, 3.33)	1.49 (0.77, 2.87)	2.26 (1.02, 5.03)*
≥ 1923 kcal/day	0.53 (0.24, 1.14)	1.34 (0.56, 3.19)	1.47 (0.63, 3.45)	1.70 (0.70, 4.14)
*p*-value for interaction	0.45	0.70	0.96	0.60

Values are odds-ratios (95% CI) per 1-SD increase.

Inverse normal transformation was applied to raw values of L-carnitine and acylcarnitines.

Acylcarnitines were summed to calculate short-, medium-, and long-chain scores.

Stratified conditional logistic regression models were adjusted for level of education (primary education or less, secondary school, university), BMI (< 25, 25–30, ≥ 30 kg/m^2^), alcohol intake (tertiles), energy intake (tertiles), diabetes, cardiovascular disease, osteomuscular disease, and physical activity (tertiles). The stratification variable was not considered as a covariate in the corresponding cases.

*MEDAS* Mediterranean Diet Adherence Screener.

*p < 0.05.

## Discussion

In this study, we found an association between levels of medium- and long-chain acylcarnitines, and a higher risk of LEFI. Since we were able to measure incident cases defined with a performance-based score, these results may help to characterize the biological pathways that lead to physical function impairment. The strongest association found among participants with lower BMI and lower energy intake suggests that these mechanisms are especially relevant for people with malnutrition, such as frail older adults.

Two previous studies have examined the association between acylcarnitines and physical function. In a cross-sectional study among 77 men  older than 70 years, Lum et al.^15^ found that a global score including 45 plasma acylcarnitines was associated with a worse score on the SPPB. As in our study, the species that most contributed to this association were the medium- and long-chain acylcarnitines. By contrast, Murphy et al.^16^ found an association of short-chain acylcarnitines with self-reported incident mobility disability in a cohort of 307 men.

The carnitine pool comprises non-esterified L-carnitine and many acylcarnitine esters^20^. Although some evidence from animal studies suggests that supplementation with L-carnitine might increase muscle mass and prevent age-associated muscle protein degradation^15^, it is unclear if dietary intake may influence endogenous carnitine levels, due to homeostatic mechanisms^10^. However, a clinical trial conducted with 70 centenarians living in Sicily^21^ found that those participants treated with L-carnitine for 6 months improved their physical and cognitive function.

On the other hand, several studies have found that elevated levels of carnitines may predict the development of diabetes^13,22^ and cardiovascular disease^14,23^. This can be due to the fact that medium- and long-chain acylcarnitines are elevated in conditions with vascular inflammation and insulin resistance^11^. Moreover, plasma levels of short-chain acylcarnitines are also higher among patients with chronic uremia, suggesting altered kidney function^24^. Both, diabetes and renal disease have been associated with frailty and mobility disability^25,26^, through low-grade inflammation, metabolic acidosis and insulin resistance, which alter intracellular energy production and muscle contraction^27^. Therefore, these mechanisms could contribute to an association between high levels of acylcarnitines and impaired physical function.

A strength of this study was the prospective design and the objective assessment of LEFI, based on the SPPB. We believe that this design has been able to select participants with a decline in lower-extremity function over the 2 years of follow-up, in comparison with participants that have remained stable. According to the definitions used, cases had to have reduced their SPPB in at least 1 point, which is clinically relevant. By contrast, controls kept an optimum level of function. Therefore, the analyses performed reflect the different concentration of acylcarnitines among participants that further decreased their physical function in comparison with those who remained with optimal function.

Although the sample size was modest, it has allowed for observing several relevant associations, and it is in line with previous metabolomics research, because of the high cost of analyzing metabolomic profiles. In addition, although a wide range of acylcarnitine species were assessed in this study, other acylcarnitines were not available to be analyzed. Further research could analyze other acylcarnitine species and their relationship with physical impairment. On the other hand, and since the stronger association found in our study between some acylcarnitines and incidence of LEFI in those with a lower BMI and a lower energy intake, future lines of research could include prospective designs considering these factors and weight loss as potential mediators of the relationship between acylcarnitines and LEFI.

In conclusion, medium- and long-chain acylcarnitines levels were associated with incident impaired physical function. This finding contributes to understand the mechanisms subyacent to the development of disability in the older people. It could also contribute to identify patients in their earlier stages of the disability process.

## Methods

### Study design

This study was performed with data from the Seniors-ENRICA (Study on Nutrition and Cardiovascular Risk) cohort in Spain^28^, which comprised non-institutionalized individuals aged ≥ 60 years at the time of baseline data collection, in 2008–2010. The Clinical Research Ethics Committee of ‘La Paz’ University Hospital in Madrid approved the study protocol (registration number: 2144). All participants in the Seniors-ENRICA cohort gave their written informed consent for taking part in the study. In 2015, a total of 1138 participants were interviewed again to update information on lifestyles and morbidity. Among them, 965 provided their written consent for the realization of a physical exam to assess LEFI and blood extraction. Blood was collected by trained nurses at the participants’ home, under standardized conditions including 12-h fasting. In 2017, a new phase of data collection was conducted among the 519 participants that remained in the study. All methods were carried out in accordance with relevant guidelines and regulations.

### Case ascertainment

Physical function was assessed using the SPPB score^29^. This test comprises three different components: gait speed, standing balance, and ability to rise from a chair. Gait speed was calculated as the time that a participant walked at habitual pace across 2.44 m. Time on the faster of two walks was used to define the scores: 1 (≥ 5.7 s), 2 (4.1 to 5.6 s), 3 (3.2 to 4.0 s), and 4 (≤ 3.1 s). In the standing balance test, participants attempted to maintain three hierarchical tandem positions: side-by-side, semitandem, and full tandem. Participants were scored 1, if they could hold a side-by-side stand for 10 s but were unable to stand in semitandem for 10 s; 2, if they stood in semitandem for 10 s but were unable to stand in full tandem for > 2 s; 3, if they stood in full tandem for 3 to 9 s, and 4 if they stood for 10 s. In addition, the ability to rise from a chair was assessed by the time required to stand up and sit down from a chair five consecutive times without helping with the arms; a score of 1 was assigned for those who spent > 16.6 s doing it, 2 for > 13.6 to 16.6 s, 3 for > 11.1 to 13.6, and 4 for ≤ 11.1 s; a value of 0 was given to those unable to do it^29^. The total SPPB score was the sum of these three components (range 0–12); a higher score reflects better lower-extremity performance. Optimal level of function was defined as a SPPB score > 9^30^. The habitual cut-off point to define impaired function in the clinical setting is ≤ 9^31^; however, we considered a cut-off point of 6 in the SPPB score to define incident cases of LEFI to improve the specificity of the test, because our population was comprised by fairly healthy community-dwelling old people^32^.

Among participants in 2015 with a SPPB > 6, those with SPPB scores ≤ 6 in 2017 were considered incident cases of LEFI in our study. A total of 43 cases of incident LEFI were identified. Mean scores ± SD for cases in 2015 and 2017 were, respectively, 9.0 ± 1.8 and 5.1 ± 1.4, showing a clear decrease in physical functioning across the follow-up. For the case–control design, we selected as controls 86 sex- and age-matched (< 65 vs. ≥ 65 y) participants (case:control ratio of 1:2), with optimum level of physical function (SPPB score > 9, mean ± SD: 10.9 ± 0.8) in 2017. SPPB mean score ± SD was 10.4 ± 1.3 for controls in 2015, which indicated that they remained with functional capacity across the follow-up. Adding cases and controls, we had a total of 129 participants.

### Acylcarnitines assessment

Plasma L-carnitine and a total of 28 acylcarnitine species were measured after solvent extraction using the 2015 blood samples, by liquid chromatography-tandem mass spectrometry (LC–MS/MS). For sample preparation, 100 µL of plasma were transferred to a microtube, and 0.5 mL of a mixture of methanol, chloroform and water (8:1:1) containing isotope labeled internal standards were added (Cambridge isotopes Free and Acyl- Carnitine set B, Tewksbury, MA, USA), and the mixture vortexed briefly. Samples were allowed to rest at 4° C for 10 m, vortexed a second time, and then centrifuged at 4° C, 14,000 RMP for 10 min. The extracts were transferred to autosampler vials for analysis. In addition, 10 µl of each sample was pooled and treated identically to samples, and the extract was analyzed along with the samples for quality control purposes. LC–MS analysis was performed on an Agilent system consisting of a 1290 UPLC module coupled with a 6490 QqQ mass spectrometer (Agilent Technologies, CA, USA; https://www.agilent.com/en/product/liquid-chromatography-mass-spectrometry-lc-ms). A 1-µL injection of each acylcarnitine metabolites was separated on an Acquity HSS-T3, 1.8 µM, 2.1 × 50 mm column (Waters, Milford, MA) maintained at 40 °C, using 10 mM ammonium acetate in water, adjusted to pH 9.9 with ammonium hydroxide, as mobile phase A, and acetonitrile as mobile phase B. Acylcarnitine transitions were monitored for the 85 Da product ion that is common to each carnitine species. The interassay coefficients of variation were ≈10%.

The list of L-carnitine and acylcarnitines measured comprises the following ones: L-carnitine (L-carn), acetyl- (C2), propionyl- (C3), butyryl- (C4), valeryl- (C5), glutaryl (C5DC), hexanoyl- (C6), octanoyl- (C8), trans-2-octenoyl- (C8:1), decanoyl- (C10), cis-4-decenoyl- (C10:1), lauroyl- (C12), trans-2-dodecenoyl (C12:1), 3-hidroxy-dodecanoyl- (C12-OH), myristoyl- (C14), cis-5-tetradecenoyl (C14:1), 3-hydroxymyristoyl- (C14-OH), palmitoyl- (C16), palmitoleoyl- (C16:1), 3-hydroxyhexadecanoyl- (C16-OH), stearoyl (C18), oleoyl- (C18:1), linoleyl- (C18:2), 3-hydroxylinoleyl- (C18:2-OH), arachidoyl- (C20), cis-11-eicosenoyl- (C20:1), 11cis,14cis-eicosadienoyl- (C20:2), eicosatrienoyl- (C20:3), and arachidonoylcarnitine (C20:4).

### Assessment of other variables

Participants in the present research were asked for their highest educational level attained. Weight and height were measured and the BMI was calculated as weight (kg) divided by squared height (m). Total energy intake (kcal/day), and alcohol consumption (g/day) were measured two years before the beginning of the present case–control study, using a validated computerized diet history^33^. The 14-item Mediterranean Diet Adherence Screener (MEDAS) was used to evaluate adherence to the Mediterranean diet^34^, with higher scores indicating a higher adherence to a healthy Mediterranean diet. Physical activity was assessed with a validated questionnaire^35^ and measured in metabolics equivalent of task (METs) given in hours/week, including the time dedicated to walk, ride a bike, gardening, climbing stairs, physical exercise, and housework. Moreover, participants reported whether they had been diagnosed with diabetes, cardiovascular disease (including heart failure, heart attack and stroke), and osteomuscular disease (including arthritis, arthrosis and hip fracture). Acylcarnitine species and covariates considered in this study were measured in 2015, while the incidence of LEFI was assessed in 2017 for defining incident cases in our case–control design.

### Statistical analyisis

One blood sample was lost during the analytical assessment; therefore, the final sample comprised a total of 128 participants: 43 of them were identified as case subjects and the remaining 85 as control subjects. Descriptive statistics were provided, separately for case and control groups, for sociodemographics and clinical characteristics. Statistical comparisons between both groups were conducted by means of unpaired *t*-tests in the case of continuous variables, while chi-square tests were used for testing potential statistical differences in terms of percentages.

Given the raw L-carnitine and acylcarnitine levels (given in µM) did not follow a normal distribution, geometric means and 95% CI were calculated for both case and control groups. Three subgroups were considered based on the type of acylcarnitines: short-chain acylcarnitines (6 acylcarnitines species), from C2 carnitine to C6 carnitine; medium-chain acylcarnitines (10 species), from C8 carnitine to C14-OH carnitine; and long-chain acylcarnitines (12 species), from C16 carnitine to C20:4 carnitine. A global score for each subgroup was created, according to the following two steps: first, plasma acylcarnitines were transformed by means of a rank-based inverse normal transformation^36^; and second, for each subgroup, a weighted sum of Z-scores was used to generate global scores in short-, medium-, and long-chain acylcarnitines, whose definition was based on the carbon chain length described above. Weights corresponded to the respective coefficients from a crude conditional logistic regression model that was fitted with each individual metabolite^37^. A higher score indicates a higher level of metabolites.

A conditional logistic regression model was used to assess the association between L-carnitine and acylcarnitine levels, and incidence of LEFI, using age-group and sex as matching variables. We calculated ORs and 95% CI per 1-SD increase in L-carnitine and in the short-chain, medium-chain, and long-chain acylcarnitine scores. Crude and adjusted models were considered. The following potential confounders were included as covariates in the different adjusted models: education level (primary education or less, secondary school, university), BMI (< 25, 25–30, ≥ 30 kg/m^2^), alcohol intake (tertiles), total energy intake (tertiles), and presence of the following chronic conditions: diabetes, cardiovascular disease, and osteomuscular disease. Tobacco consumption was not included as a confounder since the participants selected for the study were mostly non-smokers (10.9% of the participants were current smokers at the moment of the baseline interview). We added leisure time physical activity (tertiles) in a separate model because this variable was strongly related to the outcome.

As a sensitivity analysis to assess the impact of the nutritional status of the participants on the relationship between L-carnitine and the acylcarnitine scores, and incidence of LEFI, different subgroups were considered, based on the adherence to the Mediterranean diet, BMI, alcohol and energy intake. These variables were stratified according to the median value in each case. The Wald test for the interaction term, defined as the product of the L-carnitine or the acylcarnitine scores by each stratification variable, was used to examine whether the results varied across strata. Statistical significance was set at two-tailed *p* < 0.05. Analyses were conducted using Stata (version 15.1; Stata Corp., College Station).

## Data Availability

The datasets generated and analyzed during the current study are available from the corresponding author on reasonable request.

## References

[1] Tzoulaki I, Ebbels TMD, Valdes A, Elliott P, Ioannidis JPA (2014). Design and analysis of metabolomics studies in epidemiologic research: a primer on -omic technologies. Am. J. Epidemiol..

[2] Wang DD, Hu FB (2018). Precision nutrition for prevention and management of type 2 diabetes. Lancet Diabetes Endocrinol..

[3] Menni C (2013). Metabolomic markers reveal novel pathways of ageing and early development in human populations. Int. J. Epidemiol..

[4] Moaddel R (2016). Plasma biomarkers of poor muscle quality in older men and women from the Baltimore Longitudinal Study of Aging. J. Gerontol. A Biol. Sci. Med. Sci..

[5] Corona G (2014). Metabolomics biomarkers of frailty in elderly breast cancer patients. J. Cell Physiol..

[6] Fazelzadeh P (2016). The muscle metabolome differs between healthy and frail older adults. J. Proteome Res..

[7] González-Freire M (2019). Targeted metabolomics shows low plasma lysophosphatidylcholine 18:2 predicts greater decline of gait speed in older adults: the Baltimore Longitudinal Study of Aging. J. Gerontol. A Biol. Sci. Med. Sci..

[8] Wennberg AMV (2018). Plasma sphingolipids are associated with gait parameters in the Mayo Clinic Study of Aging. J. Gerontol. A Biol. Sci. Med. Sci..

[9] Longo N, Frigeni M, Pasquali M (2016). Carnitine transport and fatty acid oxidation. Biochim. Biophys. Acta.

[10] Reuter SE, Evans AM (2012). Carnitine and acylcarnitines: pharmacokinetic, pharmacological and clinical aspects. Clin. Pharmacokinet..

[11] Mihalik SJ (2010). Increased levels of plasma acylcarnitines in obesity and type 2 diabetes and identification of a marker of glucolipotoxicity. Obesity (Silver Spring).

[12] Sun L (2016). Early prediction of developing type 2 diabetes by plasma acylcarnitines: a population-based study. Diabetes Care.

[13] Guasch-Ferré M (2019). Plasma acylcarnitines and risk of type 2 diabetes in a mediterranean population at high cardiovascular risk. J. Clin. Endocrinol. Metab..

[14] Guasch-Ferré M (2016). Plasma acylcarnitines and risk of cardiovascular disease: effect of Mediterranean diet interventions. Am. J. Clin. Nutr..

[15] Lum H (2011). Plasma acylcarnitines are associated with physical performance in elderly men. J. Gerontol. A Biol. Sci. Med. Sci..

[16] Murphy RA (2019). Metabolites associated with risk of developing mobility and disability in the Health, Aging and Body Composition Study. J. Gerontol. A Biol. Sci. Med. Sci..

[17] Guralnik JM, Ferrucci L, Simonsick EM, Salive ME, Wallace RB (1995). Lower-extremity function in persons over the age of 70 years as a predictor of subsequent disability. N. Engl. J. Med..

[18] Studenski S (2011). Gait speed and survival in older adults. JAMA.

[19] Veronese N (2018). Association between gait speed with mortality, cardiovascular disease and cancer: a systematic review and meta-analysis of prospective cohort studies. J. Am. Med. Dir. Assoc..

[20] Rebouche CJ (2004). Kinetics, pharmacokinetics, and regulation of L-carnitine and acetyl-L-carnitine metabolism. Ann. N. Y. Acad. Sci..

[21] Malaguarnera M, Cammalleri L, Gargante MP, Vacante M, Colonna V, Motta M (2007). L-carnitine treatment reduces severity of physical and mental fatigue and increases cognitive funtians in centenarians: a randomized and controlled clinical trial. Am. J. Clin. Nutr..

[22] Papandreou C (2019). Plasma metabolites predict both insulin resistance and incident type 2 diabetes: a metabolomics approach within the Prevención con Dieta Mediterránea (PREDIMED) study. Am. J. Clin. Nutr..

[23] Rizza S (2014). Metabolomics signature improves the prediction of cardiovascular events in elderly subjects. Atherosclerosis.

[24] Benito S, Sánchez-Ortega A, Unceta N, Goicolea MA, Barrio RJ (2019). LC-QQQ-MS routine analysis method for new biomarker quantification in plasma aimed at early chronic kidney disease diagnosis. J. Pharm. Biomed. Anal..

[25] García-Esquinas E (2016). Serum uric acid concentrations and risk of frailty in older adults. Exp. Gerontol..

[26] Fried LF (2006). Chronic kidney disease and functional limitation in older people: health, aging and body composition study. J. Am. Geriatr. Soc..

[27] Barzilay JI (2007). Insulin resistance and inflammation as precursors of frailty: the Cardiovascular Health Study. Arch. Intern. Med..

[28] Rodriguez-Artalejo F (2011). Rationale and methods of the study on nutrition and cardiovascular risk in Spain (ENRICA). Rev. Esp. Cardiol..

[29] Guralnik JM (1994). A Short Physical Performance Battery assessing lower extremity function: association with self-reported disability and prediction of mortality and nursing home admission. J. Gerontol..

[30] Pavasini R (2016). Short physical performance battery and all-cause mortality: systematic review and meta-analysis. BMC Med..

[31] Cruz-Jentoft AJ (2010). Sarcopenia: European consensus on definition and diagnosis: report of the European working group on sarcopenia in older people. Age Ageing.

[32] Guralnik JM (2000). Lower extremity function and subsequent disability: consistency across studies, predictive models, and value of gait speed alone compared with the short physical performance battery. J. Gerontol. A Biol. Sci. Med. Sci..

[33] Guallar-Castillón P (2014). Validity and reproducibility of a spanish dietary history. PLoS ONE.

[34] Schröder H (2011). A short screener is valid for assessing Mediterranean diet adherence among older Spanish men and women. J. Nutr..

[35] Pols MA, Peeters PH, Ocké MC, Slimani N, Bueno-de-Mesquita HB, Collette HJ (1997). Estimation of reproducibility and relative validity of the questions included in the EPIC Physical Activity Questionnaire. Int. J. Epidemiol..

[36] Beasley TM, Erickson S, Allison DB (2009). Rank-based inverse normal transformations are increasingly used, but are they merited?. Behav. Genet..

[37] Wang TJ (2011). Metabolite profiles and the risk of developing diabetes. Nat. Med..

